# Prospective Comparative Analysis of Supine Versus Prone Percutaneous Nephrolithotomy in Patients with Complex Renal Stone Disease and Difficult Anatomy

**DOI:** 10.5152/tud.2024.24010

**Published:** 2024-03-01

**Authors:** Sunirmal Choudhury, Prakhar Patel, Gourab Kundu, Shahbaaz Ahmed, Malay Kumar Bera

**Affiliations:** Department of Urology Medical College and Hospital Kolkata, Kolkata, India

**Keywords:** Calcutta position, complex renal stone disease, kidney calculi, prone PCNL, stone free rate, supine PCNL

## Abstract

**Objective:**

In complex renal stone disease, few studies have shown that supine percutaneous nephrolithotomy (PCNL) is not inferior to prone PCNL. In our study, we evaluated the safety and efficacy of supine versus prone PCNL in patients with complex renal stone disease and patients with difficult anatomy.

**Methods:**

We prospectively analyzed 106 patients over 15 months from October 2022 to December 2023 and divided them as group S (Calcutta position supine arm) and group P (classical prone arm) by simple randomization. The measured data included body mass index (BMI), stone size, location of stone, number of punctures/access, tract length, bleeding, operative time, stone-free rate (SFR), length of hospital stay, and postoperative complications.

**Results:**

The operative time was 104.722 ± (34.48) versus 124.30 ± (22.67) minutes (group S vs. group P), which was significant (*P = *.01). The nephroscopy time was 89.722 ± 34.55 in group S vs. 92.212 ± 20.18 minutes, which was also significant (*P = *.01). The mean postoperative hospital stay was 3.889 ± 1.09 and 4.558 ± 1.33 days in supine and prone group (*P =* .021), respectively. Four patients in group S required re-look PCNL in comparison to 8 in group P. Overall SFR at 1 month was 76.92% and 68.51% (*P*.331), respectively in case of group S and P.

**Conclusion:**

The study revealed that supine position in Calcutta position is a viable alternative to classical prone position even in patients with complex renal stone and patients with difficult anatomy as major complications are less, SFR is higher, and need of auxiliary procedures are rare.

**Main Points**

- Staghorn calculi and multiple stones in a calyceal diverticulum or behind an infundibular stenosis are examples of complex renal stones. Additionally, stones in renal anomalies such as medullary sponge kidneys or horseshoe kidneys might be classified as complex stones. Difficult anatomy includes obese patients and patients with spinal abnormalities.
- Choudhury et al performed supine PCNL in the Calcutta position, which may be used for all kinds of stones and is carried out in a variety of body habitus and kidney morphologies.
- Studies conducted in both prone and supine positions for complex renal stones, as well as some isolated studies on difficult anatomy, have conflicting results about the advantage of prone position over supine.
- So there is a need to compare PCNL in both positions in patients with complex renal stone disease and patients with difficult anatomy and analyze the intraoperative and postoperative data.
- This study aims to compare the outcomes and complications of supine versus prone PCNL in the management of patients with complex renal stone diseases and difficult anatomical abnormality.

## Introduction 

The occurrence of kidney stones varies greatly throughout the world; rates reported are 1%-5% in Asia, 5%-9% in Europe, and 7%-15% in North America.^1^ Kidney stone prevalence in India stands at approximately 12% and is relatively more common in the northern part of India, where it is 15%.^2^ Staghorn calculi and multiple stones in a calyceal diverticulum or behind an infundibular stenosis are examples of complex renal stones. Additionally, stones in renal anomalies such as medullary sponge kidneys or horseshoe kidneys might be classified as complex stones.^3^ Difficult anatomy includes obese patients and patients with spinal abnormalities. From its initial description by Fernström and Johansson in 1976, prone PCNL has been accepted as the gold standard treatment for large kidney stones measuring ≥ 2 cm.^4^ It was instinctive to choose the prone position because of several anatomical factors, including the kidney’s posterior retroperitoneal location, the avascular line of Brödel’s, short access to the posterior calyces, the decreased risk of other viscera interposing along the working tract, and the large surface area available for puncture.^5^

A decade later, Valdivia et al^6^ described performing percutaneous nephrolithotomy (PCNL) with the patient in the supine position to lessen the encumbrances of the prone position relating to the patient, anesthesia, and operation.^6^ Later on, the Valdivia position was further improved by including a modified lithotomy position, which gave rise to the Galdakao-modified supine Valdivia position, a novel position.^7^ The main downside of this position is the restricted exposure of the flank region for renal puncture. Giusti’s modification of the Valdivia–Galdakao position came to tackle limited exposure of the flank region^8^ with nearly identical outcome to earlier studies. Choudhury et al^9^ performed supine PCNL in the Calcutta position, which may be used for all kinds of stones and is carried out in a variety of body habitus and kidney morphologies. Asymptomatic, non-infectious, and non-obstructive stone fragments (≤4 mm) seen on computed tomography (CT) scan that persist in the urinary system following the final session of any intervention (Extracorporeal shockwave lithotripsy [ESWL], Ureteroscopy [URS], or PCNL) for urinary stones are referred to as clinically insignificant residual fragments.^10,11^

Studies conducted in both prone and supine positions for complex renal stones, as well as some isolated studies on difficult anatomy, have conflicting results about the advantage of prone position over supine.^12,13^

So there is a need to compare PCNL in both positions in patients with complex renal stone disease and patients with difficult anatomy and analyze the intraoperative and postoperative data.

This study aims to compare the outcomes and complications of supine versus prone PCNL in the management of patients with complex renal stone diseases and difficult anatomical abnormality.

## Material and Methods

**Figure 1.**
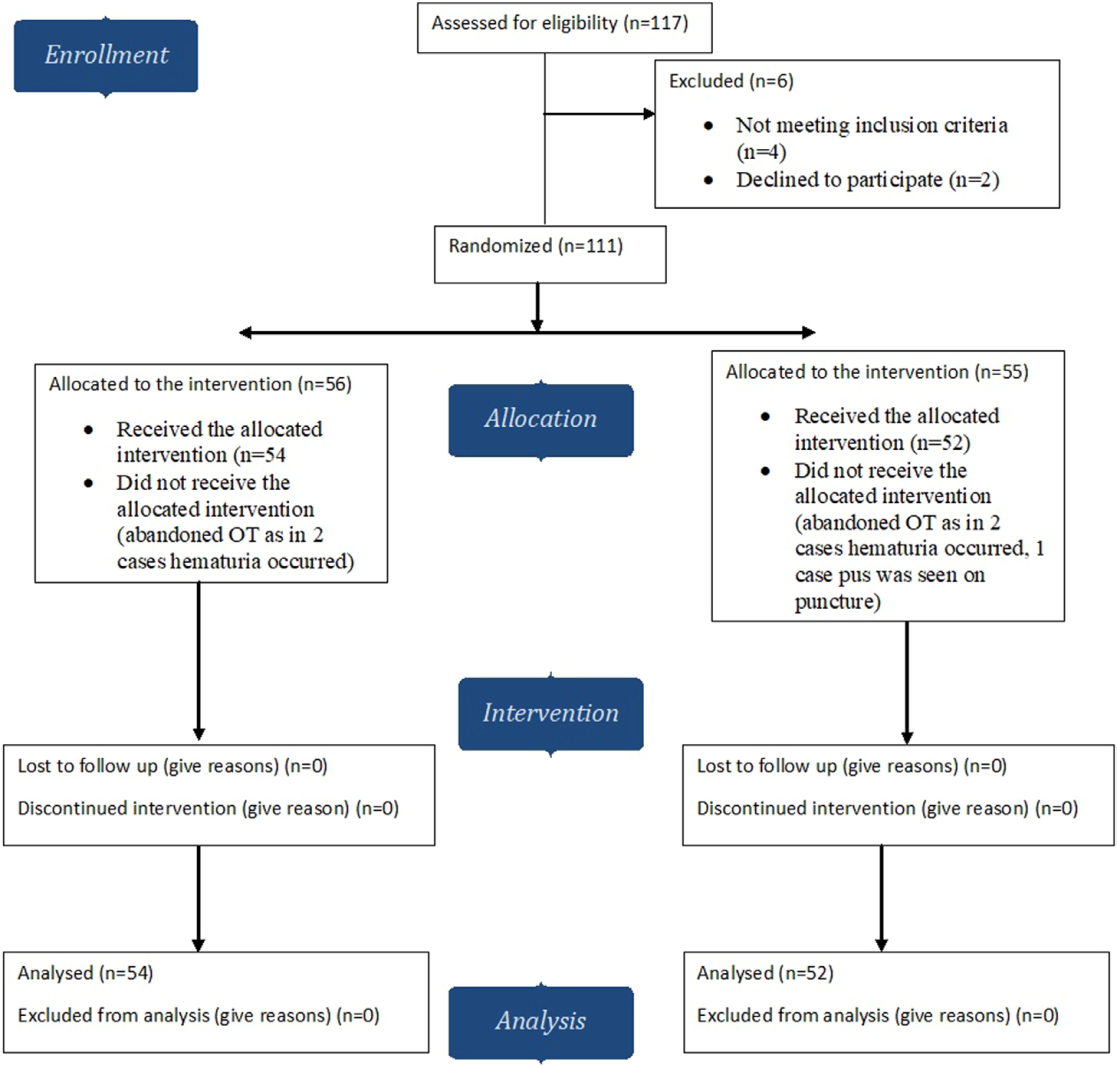
Consolidated Standards of Reporting Trials (CONSORT) diagram.

This is a prospective study in a tertiary care center in the eastern part of India for a period of 15 months from October 2022 to December 2023. Of the 117 patients enrolled in the study, 111 patients received randomly assigned interventions and 54/52 completed the study in group S/group P. After allocation, 5 patients were excluded, and none of the patients who had surgery were lost to follow-up (Figure 1).

The local ethics committee of Medical College and Hospital Kolkata approved the study protocol, and informed consent was obtained (Approval Number: MC/KOL/IEC/NON-SPON/1738/12/2022; Date: December 12, 2022). We present the study following the CONSORT guidelines.

All cases were done under general anesthesia. The puncture was done with 2 parts PCNL puncture needle. Coaxial metal dilators (Alken’s dilators) were used to dilate the tract sequentially up to 22-28 Fr sheath. Wolf nephroscope Germany (20.8 Fr/12°) and pneumatic lithotripter with 3 mm probe were used with multiple shots at 6 Hz. All patients with a diagnosis of renal stone diseases (age >18 years old) with complex stones [Guy’s stone score (GSS) of 3 or 4] on preoperative imaging (intravenous pyelography/CT scan) with or without double J stent or PCN tube with functioning ipsilateral kidney were taken.^14^ Patients with renal stone disease with difficult anatomy were also taken. We took 106 patients (54 in group S and 52 in group P) on the basis of simple randomization (1 : 1). All the patients underwent preoperatively Contrast enhanced computed tomography (CECT) kidney, ureter and bladder (KUB) with urogram to look for skin to stone distance in the supine position at a 45° angle, Hounsfield unit (HU) of stone in the center, number of stones, and any abnormal anatomy (like calyceal diverticula, horseshoe kidney (HSK), infundibular stenosis, malrotated kidney, etc.) and post-op noncontrast computed tomography (NCCT) KUB on second postoperative day to look for residual calculus, nephrostomy or stent position (if any), collections (perinephric hematoma, urinoma, hydroureteronephrosis). Follow-up after 1 month with NCCT KUB was repeated to look for residual calculus.

The operative time was defined as the time from induction of GA to closure of skin/nephrostomy placement. The nephroscopy time was considered from the introduction of nephroscope into renal system to final removal of nephroscope. The length of hospital stay was from the day of surgery to the discharge day. In our study, a single surgeon performed both procedures.

Complex renal stone disease includes patient with staghorn calculus (partial/complete), multiple stones in a patient with abnormal anatomy (infundibular stenosis/compound calyx), autosomal dominant polycystic kidney (ADPKD), calyceal diverticula, horseshoe kidney, pelvic kidney or other malrotation abnormality, and duplex system with calculus. Difficult anatomy in patients includes kyphoscoliosis, polio, and obesity.

**Figure 2.**
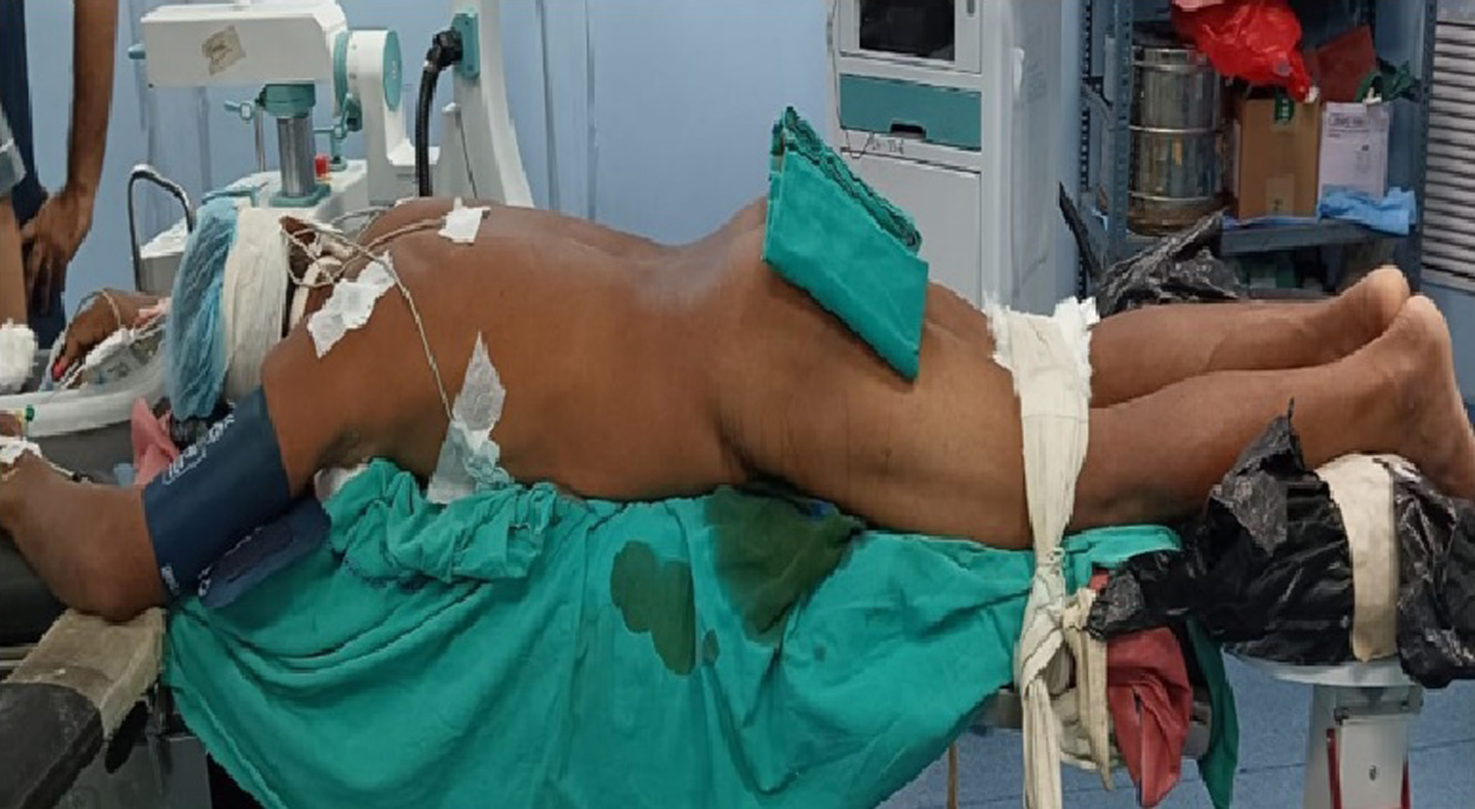
Classical prone position.

In group P after ureteral catheterization, the patient is shifted to the prone position and fixed to a 14 Fr Foley catheter. Using the triangulation or bull’s-eye method, a 15 cm long, 18G piercing needle was used for the initial puncture. A Terumo guide wire of size 0.035″ is passed and serial dilatation is done over metallic guide rod up to 22-28 Fr to allow Amplatz sheath of same size. Using a pneumatic lithoclast, stone is broken apart and then extracted using alligator forceps. In each case, a double J stent of 5 Fr/26 cm was inserted, either with or without a nephrostomy tube (Figure 2).

**Figure 3.**
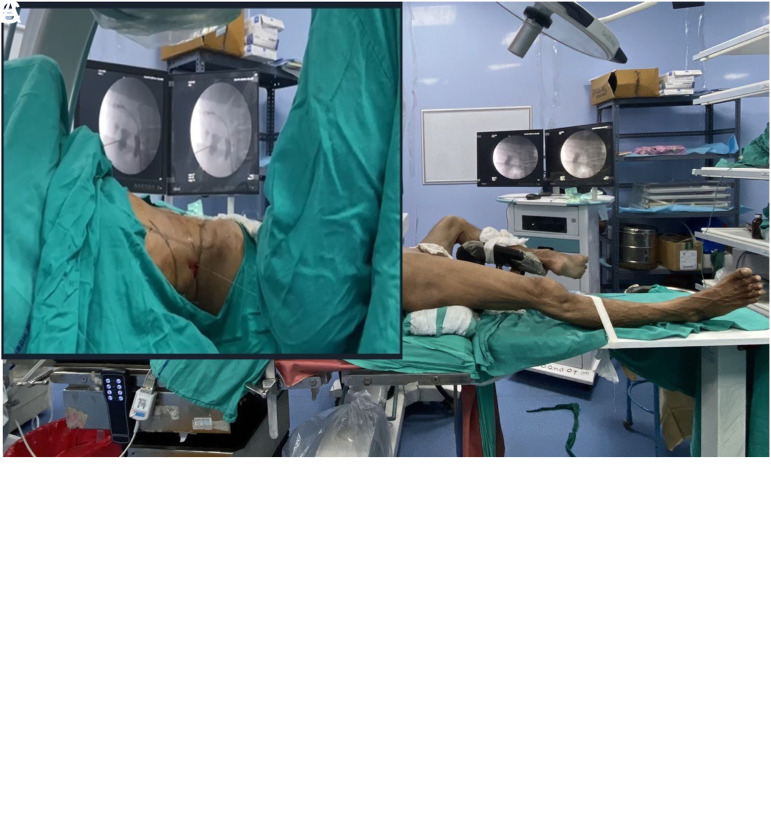
(A) Calcutta position in patient with kyphoscoliosis with right duplex system. (B) X-ray showing kyphoscoliosis with right renal calculus. (C) Initial puncture done using triangulation technique in middle calyx.

In group S the iliac crest, 12th rib, and posterior axillary line were surface marked while the patient was in a standing position. Patients were placed in the modified Calcutta supine posture following ureteral catheterization. The anesthetized patient was positioned at the lateral edge of the operating table in a fully supine posture, with the detachable leg plate supporting the ipsilateral leg of the PCNL side. With support from a single stirrup, the contralateral leg was flexed and abducted at the hip joint. Two self-made bolsters, 1 below the hip and 1 below the shoulder, were positioned horizontally against the torso. To prevent any stretch over, the ipsilateral arm was brought across the chest to the contralateral side and held with a stirrup to avoid any stretch over the brachial plexus. A tilt of 10°-15° was achieved by the bolster below the shoulder only. Initial puncture is done with a 15 cm long 18 G needle in the triangulation technique. The rest of the procedure is the same as that of prone PCNL.^9^ (Figure 3)

### Statistical Analysis

The entire data collected in an Excel sheet will be thoroughly evaluated using analysis of variance test, chi-square test, and Student-*t* test. Analysis of variance test will be used to compare mean between the 2 groups. Comparative variables will be compared using the student-*t* test. *P *< .05 will be considered statistically significant. All analyses were carried out by using Statistical Package for the Social Sciences (SPSS) version 16.0 (SPSS Inc.; Chicago, IL, USA).

## Results

The mean age group in group S was 44.407 ± 11.480 (range = 25-67 years) while in group P was 39.846 ± 10.719 years (range = 22-65 years). Other demographics like body mass index, American Society of Anesthesiologists (ASA) score, laterality of stones, stone burden, HU of stone density were all comparable with insignificant *P*-values (Table 1). Group S had a shorter operative time (104.722 ± 34.48 vs. 124.327± 22.67 minutes), which was found to be significant. The nephroscopy time was 89.722 ± 34.55 in group S vs. 92.212 ± 20.18, which was also significant (Table 2). Tract length was more in group S when compared to group P (*P* = .000*).

**Figure 4.**
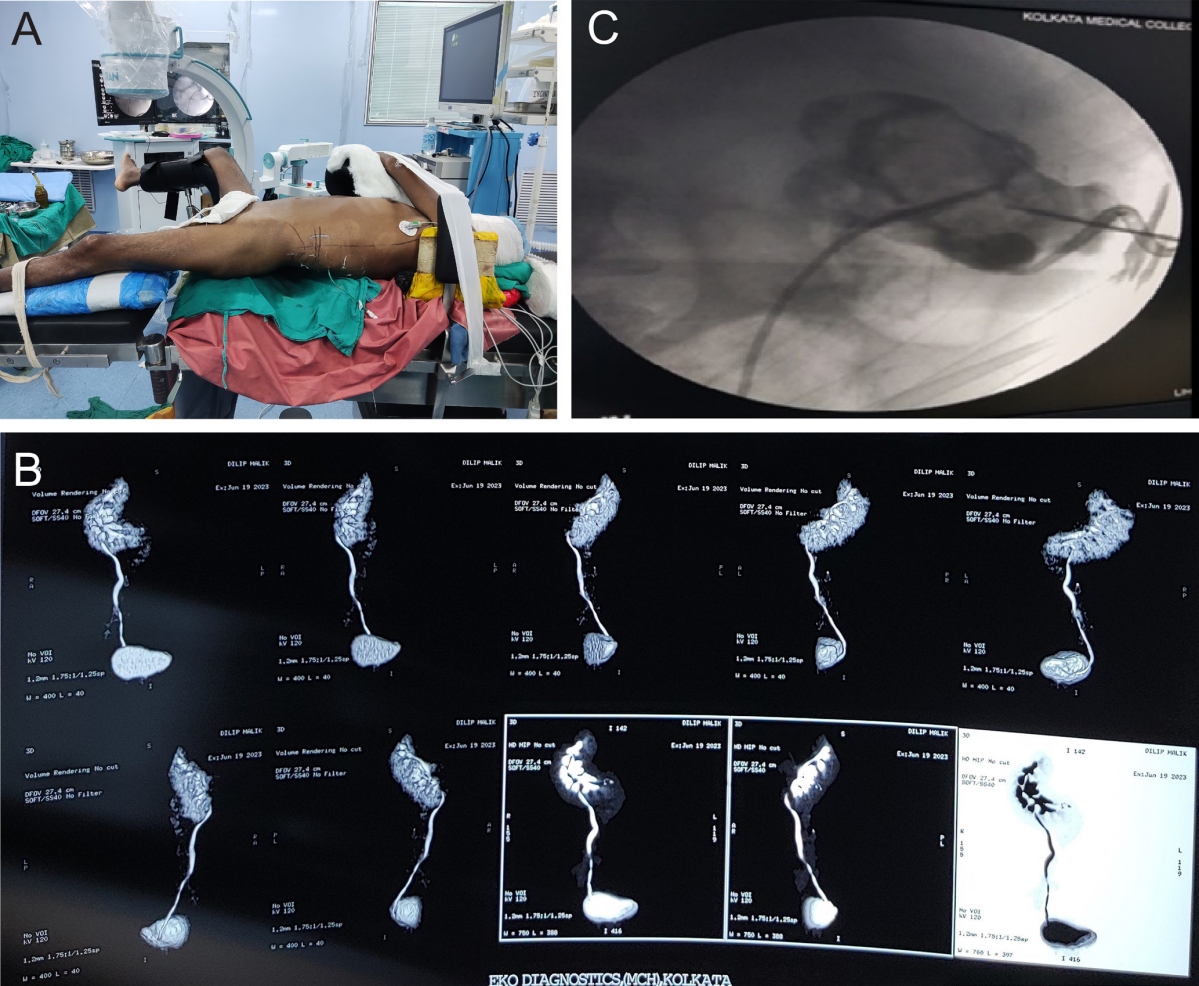
(A) Calcutta position in horseshoe kidney patient. (B) Preoperative computed tomography images of patients with horseshoe kidney. (C) Intraoperative fluoroscopic images of the patient.

**Figure 5.**
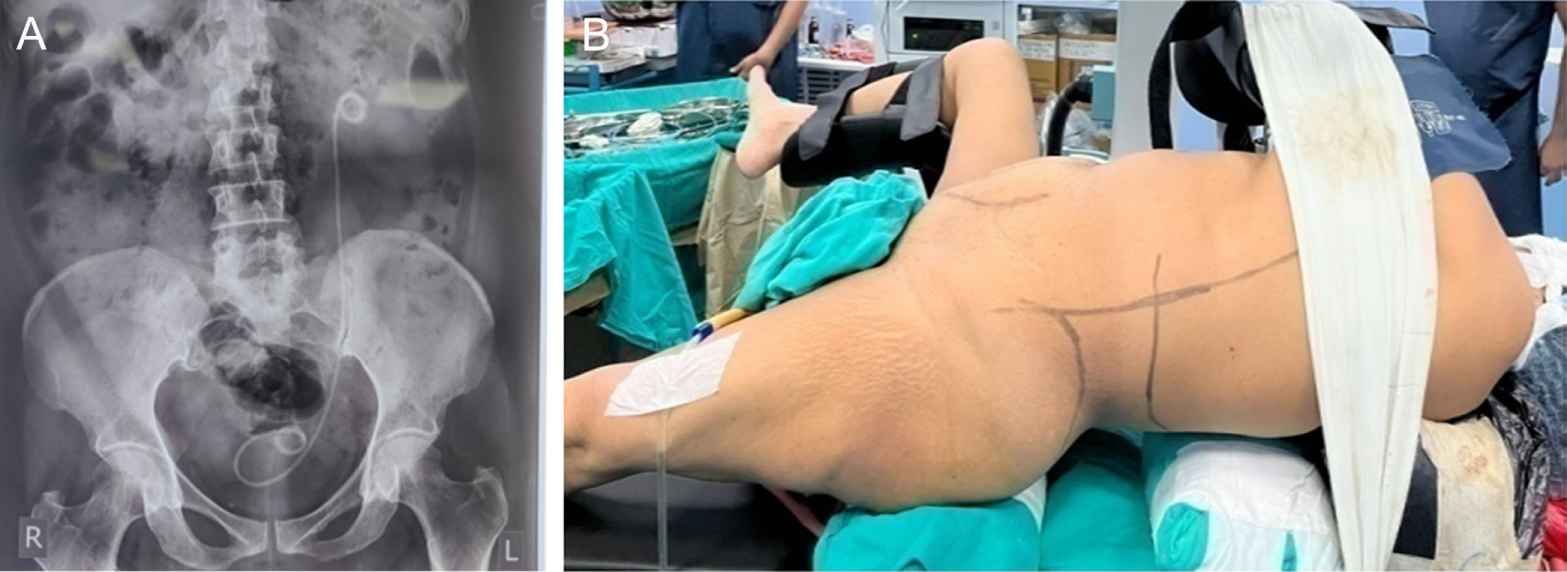
(A) Preoperative x-ray showing left encrusted DJ stent with renal calculus as well as bladder calculus in an obese patient. (B) Calcutta-modified supine position with markings.

Stone-free rate was higher in group P in diverticular stone, staghorn stone, while group S was higher in obese, ADPKD, kyphoscoliosis, and HSK although none of these data were significant (Figure 3A, 3B, 3C, Figure 4A, 4B, 4C, and Figure 5A, 5B) In duplex system and malrotated kidney, both positions had equal SFR. One case of poliomyelitis was operated in the supine group and was stone free. Overall SFR at 1 month was 76.92% and 68.51% (*P*-value 0.331), respectively in the case of groups S and P (Table 3).

Duration of hospital stay was 3.889 ± 1.09 days in group S vs. 4.558 ± 1.33 in group P, which was significant (Table 4).

The overall complication rate was 14.15% according to Modified Clavien–Dindo classification for PCNL.^15^ Group P had a higher rate of Clavien ≥3 complications. None of the patients in group S required ESWL on follow-up compared to the 4 patients in group P. The fall in post-op hemoglobin was not significant in both groups (in group S it is 1.841 vs. In group P 2.058), but group P required 3 Blood transfusion (BT) compared to 1 in group S. Rise in serum creatinine in group S is 0.183 vs. 0.149, which was insignificant.

## Discussion

Prone PCNL is an accepted procedure that dates back many years. According to Valdivia et al,^6^ supine PCNL has proven to be an effective alternative since it was first introduced.^5^ However studies comparing supine versus prone PCNL in complex renal stone disease with difficult anatomy are scarce. Ahn et al^16^ showed that prone PCNL done in complex renal stone disease has an overall high success rate (100%) with a low complication rate (15.4%) done in 69 patients. Furthermore, renal entrance behind the stone is as viable and safe as approaching via a dilated renal calyx, as demonstrated by Ahn et al^16^ using the triangulation technique. Perrella et al^12^ conducted a randomized research study which demonstrated that the positioning of the patient during PCNL for complex kidney stones had no effect on the success rates. Supine may be linked to a lower incidence of high-grade complications than prone; however, further research is needed on this subject.^12^ No bowel injury was reported in our study. A 0.2%-0.3% rate of colonic perforation has been recorded in other investigations; however, these reports primarily concerned patients with complicated anatomy, such as a horseshoe kidney.^17,18^

Choudhury et al^9^ demonstrated that supine PCNL in the Calcutta position is a safe and efficient approach for managing nephrolithiasis. Together with the advantages of supine PCNL, these benefits also include enhanced nephroscope and C-Arm maneuverability. Supine PCNL in the Calcutta position was tested in multiple scenarios, and findings were similar.

Our studies suggest that a significant difference between the operative time, nephroscopy time, and overall complications is in favor of supine, which corresponds to other studies as well.^19,20^

The SFR was higher in supine position as compared to prone position (76.92% and 68.51%). Our study contrasts with the Clinical Research Office of the Endourological Society (CROES) study, in which prone PCNL had a higher SFR. Because the CROES study’s cases were not randomized and their success was not standardized, thus it has a reduced evidence level.^21^

Mean tract length was higher in group S owing to percutaneous puncture comes from a more lateral position on the patient’s flank than in prone position—this may increase the tract length of supine PCNL. On the other hand, tract length is often shorter during prone PCNL. The anterior abdominal wall’s greater pliability than the posterior abdominal wall could potentially be a contributing factor in this. In the prone position, the anterior abdominal wall, which is more flexible, transfers the pressure of the bed on the kidneys, causing the kidneys to move less freely and the percutaneous tract to get shorter.^22,23^

The overall SFR at 1 month in our study was 72.6%, which is comparable with Osther et al’s^13^ study on PCNL in patients with renal anomalies.

Overall complications were higher in prone PCNL (21.15% vs. 7.40%). Perioperative bleeding requiring quitting the operation was the major complication seen in both groups (prone > supine). Postoperative fever was also seen more in prone PCNL, which can be explained by higher intra-renal pressure but has yet to be proven.^24^ The length of hospital stay was higher in prone PCNL, which matches with the study by Perrella et al.^12^

A major limitation of our study was that only a small number of patients were evaluated and the period of study being only 15 months adds to the above limitation.

Our study shows supine PCNL in the Calcutta position is safe to manage complex renal stones with difficult anatomy. It has many advantages over prone PCNL like shorter operative time, nephroscopy time, and overall complications are less. The need for blood transfusion is less, the need of auxiliary procedures is rare, and duration of hospital stay is less. However, prone PCNL still finds its place in patients having staghorn calculus and diverticular stone. Overall SFR is similar in both prone and supine PCNL.

## Figures and Tables

**Table 1.** Demographic, Biochemical, and Radiological Data of Patients with Complex Renal Stone Disease and Difficult Anatomy

	Group S	Group P	*P*
Patients (n)	54	52	
Male/female (n)	32/22	29/23	.716
Years (mean ± SD)	44.407 ± 11.4801	39.85 ± 10.72	.506
BMI, kg/m^2^ (mean ± SD)	24.176 ± 2.8330	23.38 ± 2.28	.111
American Society of Anesthesiologists score, I/II/III (n)	22/26/06	18/29/05	.734
Previous stone-related surgery (n)	10	12	*–*
Laterality right /left (n)	32/22	25/27	.248
Solitary kidney (n)	01	02	*–*
Stone burden, mm^3^ (mean ± SD)	690.759 ± 737.276	685.577 ± 240.232	.961
HU stone density (mean ± SD)	1072.037 ± 294.84	949.962 ± 217.19	.062
Pre-op hemoglobin, gm/dL (mean ± SD)	12.426 ± 1.469	12.308 ± 1.639	.736
Pre-op creatinine, ng/dL (mean ± SD)	1.06 ± 0.25	0.94 ± 0.22	.415

BMI, body mass index.

**Table 2.** Operative Variables of Patients with Complex Renal Stone Disease and Difficult Anatomy

	Group S	Group P	*P*
Patients (n)	54	52	
Punctures (n) P50% P25%-P75%	2 1-4	1 1-4	–
No. of accesses 1 More than 1	36 18	31 21	.451
First pole accessed (n) Lower Middle Upper	37 13 4	30 14 8	.356
Supracoastal access(%) (n) Tenth intercoastal access Eleventh intercoastal access	0 1	0 6	–
Staged procedure (%) (re-look PCNL) (n)	8	11	–
Operative time (mean ± SD)	104.722 ± 34.48	124.327 ± 22.67	.000*
nephroscopy time (mean ± SD)	89.722 ± 34.55	92.212 ± 20.18	.000*
Mean tract length (cm)	10.144	9.923	.000*

PCNL, percutaneous nephrolithotomy.

*P < .05 (significant).

**Table 3.** Operative Outcome

	Group S	Group P	*P*
Diverticular stone SFR at 1 month	3 2/3 = 67%	3 3/3 = 100%	.273
Staghorn calculus SFR at 1 month	24 16/24 = 66.66%	26 19/26 = 73.07%	.852
Kyphoscoliosis SFR at 1 month	3 2/3 = 66.67%	2 1/2 = 50%	.709
Obesity SFR at 1 month	14 12/14 = 85.71%	10 07/10 = 70%	.350
HSK SFR at 1 month	3 2/3 = 66.67%	5 3/5 = 60%	.527
ADPKD SFR at 1 month	3 3/3 = 100%	3 2/3 = 66.67%	.273
Duplex kidney SFR at 1 month	1 1/1 = 100%	1 1/1 = 100%	* – *
Poliomyelitis SFR at 1 month	1 1/1 = 100%	0	*–*
Malrotated kidney SFR at 1 month	2 1/2 = 50%	2 1/2 = 50%	1
Overall SFR	40/52	37/54	.331
Post-op hemoglobin	10.585 ± 1.25	10.250 ± 1.23	.766
Post-op creatinine	1.243 ± .3294	1.089 ±.1269	.056
Length of hospital stay (days)	3.889 ± 1.09	4.558 ± 1.33	.021*

*P < .05 = significant.

**Table 4.** Complications (modified Clavien–Dindo Classification for percutaneous nephrolithotomy)

	GROUP S	GROUP P	*P*
Patients (n)	54	52	
Overall complications (n)	04 (7.40%)	11 (21.15%)	.042*
Major complications (n)	02	03	.616
Clavien-Dindo’s classification (n) I II IIIa IIIb	01 01 1 1	05 03 01 02	.109

*P < .05 (significant).
